# Second-generation inhibitors of Bruton tyrosine kinase

**DOI:** 10.1186/s13045-016-0313-y

**Published:** 2016-09-02

**Authors:** Jingjing Wu, Christina Liu, Stella T. Tsui, Delong Liu

**Affiliations:** 1 Department of Oncology, The first Affiliated Hospital of Zhengzhou University, Zhengzhou, 450052 China; 2 Weinberg College of Arts and Sciences, Northwestern University, Evanston, IL 60208 USA; 3 SUNY Stony Brook University, Stony Brook, NY 11794 USA

## Abstract

Bruton tyrosine kinase (BTK) is a critical effector molecule for B cell development and plays a major role in lymphoma genesis. Ibrutinib is the first-generation BTK inhibitor. Ibrutinib has off-target effects on EGFR, ITK, and Tec family kinases, which explains the untoward effects of ibrutinib. Resistance to ibrutinib was also reported. The C481S mutation in the BTK kinase domain was reported to be a major mechanism of resistance to ibrutinib. This review summarizes the clinical development of novel BTK inhibitors, ACP-196 (acalabrutinib), ONO/GS-4059, and BGB-3111.

## Background

### Bruton tyrosine kinase

Bruton tyrosine kinase (BTK) was initially implicated in the pathogenesis of X-linked agammaglobulinemia [1–4]. The gene encoding the BTK molecule was isolated in 1993 and was named independently at the time as B cell progenitor kinase and agammaglobulinemia tyrosine kinase [5, 6]. The BTK gene is located on the X chromosome in the region Xq21.3-22.1. The gene contains 19 exons and the open reading frame has 1977 nucleotides. BTK is a 76-kDa polypeptide with 659 amino acid residues.

#### BTK functions

**Fig. 1:**
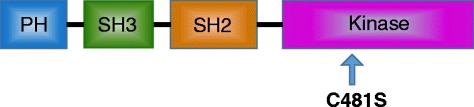
The structure of Bruton tyrosine kinase (BTK). BTK has a pleckstrin-homology (PH) domain, SH3 and SH2 (src-homology) domains, and a kinase domain. The BTK polypeptide has 659 amino acid residues with an approximate molecular weight of 76 kDa. The C481S mutation in the kinase domain mediates resistance to ibrutinib

BTK is expressed in the cells of all hematopoietic lineages except for T and plasma cells [7]. It is a cytoplasmic tyrosine kinase in the Tec family [8]. Like other Tec family members, BTK has a PH (pleckstrin-homology) domain, SH3 and SH2 (src-homology) domains, and a carboxyl kinase domain (Fig. 1). This tyrosine kinase lies downstream of the B cell antigen receptor (BCR) [9]. Upon activation of BCR, BTK becomes activated through interacting with the partner molecules through the PH and SH domains [10, 11]. This in turn leads to calcium release [8, 12]. BTK is a critical effector molecule and is involved in all aspects of B cell development, including proliferation, maturation, differentiation, apoptosis, and cell migration [13]. When the BTK gene was knocked out in a mouse model, a reduced number of mature B cells along with severe IgM and IgG3 deficiency were observed [14]. BTK is critical in the initiation, survival, and progression of B cell lymphoproliferative disorders [15–17].

### Ibrutinib: the first-generation BTK inhibitor

Targeting novel biomarkers that are driver molecules regulating cancer cell growth and differentiation has revolutionized drug development for cancer therapy [18–24]. Novel agents targeting biomarker molecules in lymphocytes are revolutionizing treatment of lymphoid malignancies [25–33]. Since BTK is a critical effector molecule for B cell development and plays a major role in lymphomagenesis, BTK inhibitors have been investigated as potential treatments [11, 34–37]. To date, ibrutinib remains the only BTK inhibitor approved for several lymphoproliferative malignancies [38–40].

Ibrutinib is the first-in-class, highly potent small molecule inhibitor that selectively binds to cysteine 481 residue in the allosteric inhibitory segment of BTK kinase domain. The compound irreversibly abrogates the full activation of BTK by inhibiting its autophosphorylation at tyrosine residue 223 [41]. Ibrutinib (imbruvica) has been approved for the treatment of chronic lymphocytic leukemia (CLL), mantle cell lymphoma (MCL), and Waldenstrom’s macroglobulinemia [11, 35, 36, 38–40, 42–46]. However, untoward effects, such as bleeding, rash, diarrhea and atrial fibrillation have been observed and attributed in part to its off-target effects on the epidermal growth factor receptor and the Tec family proteins other than BTK [8, 43, 44, 47–53]. In addition, resistance to ibrutinib has been observed [54, 55]. As a result, second-generation BTK inhibitors are being developed.

### Resistance mechanisms for ibrutinib

The estimated progression-free survival (PFS) rate among relapsed/refractory CLL patients treated with ibrutinib was reported to be 75 % at 26 months [38]. The mechanisms of acquired resistance to ibrutinib are under active investigation [54–56].

In one case report, a CLL patient developed resistance after 21 months on ibrutinib at a dose as high as 840 mg daily [55]. Through sequencing RNA from pre- and post-treatment samples, a thymidine-to-adenine mutation at nucleotide 1634 of the BTK complementary DNA (cDNA) was discovered. This led to a substitution of serine for cysteine at residue 481 (C481S) (Fig. 1). Ibrutinib forms a covalent bond with the sulfhydryl group of C481 of BTK and irreversibly inhibits the kinase activity of BTK [41]. The new amino acid residue S481 prevents ibrutinib from covalently binding to the BTK mutants, converting irreversible inhibition of the BTK to reversible inhibition. When the phosphorylation at tyrosine residue 223 was studied, the IC50 (half-maximal inhibitory concentration) of ibrutinib changed to 1006 nM on C481S mutant BTK from 2.2 nM on non-mutant BTK [55]. The C481S mutation was below the detectable level in ibrutinib-naïve patients, suggesting that this mutant clone was selected out through BTK inhibition by ibrutinib [57]. The same C481S BTK mutation was also found to be responsible for acquired resistance to ibrutinib in MCL [56, 58].

In addition to the C481S mutation, three distinct mutations in PLCγ2 were found in two CLL patients who became resistant to ibrutinib [54]. Two mutations in PLCγ2, R665W and L845F, could lead to a gain-of-function. Since PLCγ2 lies immediately downstream of BTK, these mutants could therefore bypass the inactive BTK and allow autonomous B cell receptor activity despite the inactive BTK. In an update, two of six patients with Richter transformation after ibrutinib therapy had the BTK C481S mutation, while 100 % patients (10/10) with progression but no Richter transformation had either one or both BTK C481S and PLCγ2 mutations [54]. None of the mutations were present in any of the patients with prolonged lymphocytosis on ibrutinib therapy.

Approximately 32 % of MCL patients had primary resistance to ibrutinib since the response rate of MCL to ibrutinib was 68 % [40]. This suggests that additional mechanisms of resistance exist. It was demonstrated that inhibition of ERK1/2 and AKT correlated with cellular response to BTK inhibition in vitro and in primary tumor samples [58]. Taken together, primary ibrutinib resistance in MCL is not mainly caused by ineffective ibrutinib inhibition of BTK but rather involves PI3K-AKT activation. In addition, transcriptome sequencing displayed recurrent mutations in *TRAF2* or *BIRC3* in 15 % of the 165 patient samples [59]. These genetic lesions in the alternative NF-kB pathway exposed another mechanism of primary ibrutinib resistance in MCL.

In patients with Waldenstrom’s macroglobulinemia (WM), MYD88 and CXCR4 mutations have been shown to be associated with clinical response to ibrutinib [46, 60, 61]. The effect of MYD88 and CXCR4 mutations on outcomes of ibrutinib in 63 patients with WM was reported [39, 46]. Results indicate that patients with MYD88^L265P^CXCR4^WT^ have the highest response rate (100 % overall response rate). BCL2 can protect against ibrutinib triggered apoptosis regardless of CXCR4^WHIM^ mutation status [60, 62], supporting the use of BCL2 inhibitor in refractory B cell malignancies [19].

There is, however, no relationship between MYD88 mutation status and ibrutinib response in patients with diffuse large B cell lymphoma (DLBCL), particularly the ABC subtype. Instead, mutations that affect the signaling of B cell and T cell receptors, such as CD79A/B and CARD11 may be responsible for lower response to ibrutinib [39, 63, 64].

### Second-generation BTK inhibitors

The emerging resistance to and off-target side effects of ibrutinib have led to active development of second-generation and more specific BTK inhibitors, such as ACP-196, ONO/GS-4059, and BGB-3111.

### ACP-196

ACP-196, also known as acalabrutinib, is a novel irreversible second-generation BTK inhibitor [34, 37]. It is more potent and selective than ibrutinib with reduced off-target side effects. As shown through IC50 determinations on nine kinases with a cysteine residue in the same position as BTK, ACP-196 had virtually no inhibition on kinase activities of EGFR, ITK, TEC, etc. [37, 65, 66].

A phase 1/2, multicenter, open-label, and dose-escalation clinical trial on ACP-196 (NCT02029443) has been underway in relapsed and refractory CLL patients. In the last update, 61 patients with relapsed CLL were enrolled [37]. In the phase 1 portion of this study, patients were treated with ACP-196 at an increasing dose of 100 to 400 mg once daily, while in the phase 2 expansion portion, 100 mg twice daily was given. The median follow-up time was 14.3 months (range 0.5–20), the overall response rate (ORR) was 95 %, with 85 % partial response (PR), 10 % PR, and 5 % SD (stable disease). Among them, patients with chromosome 17p13.1 deletion had 100 % ORR. The most common adverse events were headache, diarrhea, and weight gain, without dose-limiting toxicities, and no cases of atrial fibrillation and Richter’s transformation.

Currently, a phase 3 study (NCT02477696) directly comparing ACP-196 with ibrutinib in high-risk patients with relapsed CLL has commenced. In addition, multiple trials of ACP-196 on other hematological malignancies and solid tumors are underway [34].

### ONO/GS-4059

ONO/GS-4059 is another highly potent and more specific BTK inhibitor. Its anti-tumor activities were studied in preclinical models [67] and in the clinical trials for the treatment of B cell malignancies [68–73].

In an ABC-DLBCL cell line (TMD-8) xenograft model, the effects of ONO/GS-4059 on gene transcription in vivo were analyzed [67]. The results indicated that ONO/GS-4059 affects a core set of genes that contain nine down-regulated and eight up-regulated genes in a dose-dependent manner. Among these, CXCL-10 is the most down-regulated gene by ONO/GS-4059 and is involved in the pathological processes of human disorders, such as infectious diseases and inflammatory and autoimmune diseases as well as cancer. This study confirmed the profound anti-proliferative activity of ONO/GS-4059 by inhibiting BTK in the TMD-8 model.

The first-in-human phase I study of ONO/GS-4059 was on relapsed/refractory B cell malignancies (NCT01659255) [70]. The efficacy and safety data on 90 evaluable patients (CLL *n* = 28, MCL *n* = 16, DLBCL *n* = 35, FL *n* = 5, WM *n* = 3, MZL *n* = 2, and SLL *n* = 1) were reported [68–70, 72]. The dose-escalating 3 + 3 cohorts ranged from 20 mg to 600 mg once daily, and twice-daily regimens had doses of 240 mg and 300 mg. In the CLL group, 96 % (24/25) of patients had objective responses within the first 3 months of therapy. Rapid responses in the lymph nodes were noted with concurrent lymphocytosis [72]. In the MCL group, 92 % (11/12) of patients responded to ONO/GS-4059 (six PR and five complete responses (CRs)/Cru). In non-germinal center DLBCL, 35 % (11/31) of patients responded with 2 confirmed CR, 1 CRu, and 8 PR. In contrast to CLL and MCL, responses of DLBCL were much less durable. Notably, CLL and MCL patients with a chromosome 17p deletion and/or TP53 mutation as well as those following allogeneic stem cell transplantation responded rapidly. The pharmacokinetics of ONO/GS-4059 showed rapid absorption and elimination with a half-life of 6.5 to 8 h. The BTK occupancy in the peripheral blood was maintained for at least 24 h across all dose levels. Most importantly, ONO/GS-4059 was found to be well tolerated in all groups. There was no maximal tolerated dose (MTD) reached in the CLL group. In the lymphoma cohort, 480 mg once daily was the MTD. Drug-related hematoma was reported in one patient. Atrial fibrillation was observed, but it was reported to be not drug-related [72]. In the kinomescan study, ONO/GS-4059 was found to have significantly weaker activity on TEC kinase [72]. Therefore, ONO/GS-4059 has a favorable safety profile along with preliminary efficacy in patients with relapsed/refractory B cell malignancies.

Further investigations of ONO/GS-4059 are ongoing to ascertain its advantages in combination therapies. Combination of idelalisib and ONO/GS-4059 synergistically inhibited the growth of a subset of DLBCL and MCL cell lines [71]. This combination led to more significant growth inhibition of the A20 mutant TMD8 cells than single agent idelalisib. This suggested that the combination therapy may overcome some mechanisms of resistance in the BTK signaling pathway. In addition, Jones et al. investigated the potential activity of combinations of the B cell receptor pathway inhibitors, entospletinib, ONO/GS-4059, and idelalisib, with the BCL2 inhibitor ABT-199 in primary CLL cells [73–76]. Results showed that their combination synergistically increased the apoptosis in these primary CLL cells and achieved the maximal levels of apoptosis. These data support clinical investigation of these combinations in patients with CLL.

### BGB-3111

BGB-3111 is another more selective BTK inhibitor with superior oral bioavailability, higher BTK specificity than ibrutinib [24, 77].

In preclinical studies, BGB-3111 showed more restricted off-target activities against a panel of kinases, including ITK. Due to the weaker activity on ITK, BGB-3111 was at least 10 times weaker than ibrutinib in inhibiting rituximab-induced ADCC activity. Both in the REC-1 MCL and ABC subtype DLBCL (TMD-8) xenograft models, BGB-3111 induced dose-dependent anti-tumor effects and demonstrated superior efficacy in comparison with ibrutinib. Toxicity study in rats indicated that BGB-3111 was very well tolerated, and the MTD was not reached when it was dosed up to 250 mg/kg/day [77]. These preclinical data showed that BGB-3111 is a highly selective and potent BTK inhibitor.

The first-in-human, open-label phase 1 trial of BGB-3111 is ongoing as a modified 3 + 3 dose-escalation design (40, 80, 160, 320 mg PO QD; 160 mg PO BID) in patients with advanced B cell malignancies [24]. The pharmacokinetics, efficacy and safety of BGB-3111 were assessed in this study. At the last update from the 2015 ASH annual meeting, 25 patients were enrolled in five cohorts: 40 mg (*n* = 4), 80 mg (*n* = 5), 160 mg (*n* = 6), 320 mg (*n* = 6) QD, and 160 mg BID (*n* = 4). Sixty-four percent (16/25) of patients had objective responses, including 1 CR and 6 SD. There were no drug-related adverse events (AEs), no dose-limited toxicities (DLT) reported yet, and the MTD was not reached. The preliminary phase 1 results suggested that the selective BTK inhibitor BGB-3111 is clinically active and tolerable. However, the study report was preliminary, and its toxicity profile and clinical efficacy remain to be determined.

## Conclusions

Second-generation and more selective BTK inhibitors, ACP-196, ONO/GS-4059, and BGB-3111, are being evaluated clinically. These compounds have fewer off-target effects and are more potent than ibrutinib (Table 1). Ibrutinib has been shown to be well tolerated and effective in combinations with chemotherapy regimens [78, 79]. The more selective ACP-196 and ONO/GS-4059 are being investigated in combinations with active agents in lymphoma therapy. With the advances in bispecific antibodies [80–83], antibody drug conjugates [84, 85], immune checkpoint blockers [86, 87], and CAR-T for cancer immunotherapies [88–90], further investigation of combinations with these agents will lead to less toxic and more targeted therapeutic regimens for B cell malignancies.

**Table 1:** Comparison of ibrutinib with second- generation BTK inhibitors

	Ibrutinib	ACP-196	ONO/GS-4059	BGB-3111
Target	BTK	BTK	BTK	BTK
Major off-targets	EGFR, ITK, TEC	Minimal	TEC (weak)	ITK (weak)
Activity on C481S	No	Yes	Yes	Yes
Platelet inhibition	Yes	No	NA	NA
Atrial fibrillation	Observed	Not observed	Observed^a^	NA
Approved indications	CLL/SLL, MCL,WM	None	None	None

*BTK* Bruton tyrosine kinase, *NA* not available /reported, *CLL* chronic lymphoid leukemia, *SLL* small lymphoid leukemia, *MCL* mantle cell lymphoma, *WM* Waldenstrom’s macroglobulinemia

^a^The atrial fibrillation was not thought to be drug-related

## Abbreviations

AE: adverse event
BTK: Bruton tyrosine kinase
CR: complete response
ORR: overall response rate
PR: partial response
SD: stable disease
DLT: dose-limiting toxicity
MTD: maximal tolerated dose
